# The Effect of 30% or More Volume Reduction Achieved by Surgical Ventricular Reconstruction for Ischemic Cardiomyopathy: A Systematic Review

**DOI:** 10.1016/j.shj.2024.100390

**Published:** 2025-01-28

**Authors:** Harun Osmani, Paulien Christine Hoefsmit, Romy R.M.J.J. Hegeman, George Louis Burchell, Jolanda Kluin, Patrick Klein, Reinier Zandbergen

**Affiliations:** a Department of Cardiothoracic Surgery, Amsterdam University Medical Center, Amsterdam, The Netherlands; b Medical Library, Vrije Universiteit Amsterdam, Amsterdam, The Netherlands

**Keywords:** Heart failure, Ischemic cardiomyopathy, Left ventricular remodeling, Surgical ventricular reconstruction, Mitral regurgitation

## Abstract

**Background:**

Ischemic cardiomyopathy, commonly attributable to coronary artery disease, leads to ventricular remodeling. Surgical ventricular reconstruction (SVR) aims to reverse this by restoring cardiac morphology. This review evaluates SVR's influence on ventricular volume reduction, comparing <30% to >30% reduction effects on left ventricular ejection fraction (LVEF) and patient outcomes.

**Methods:**

A systematic search following Preferred Reporting Items for Systematic Reviews and Meta-Analysis guidelines was performed in PubMed, Embase, Web of Science, and the Cochrane Collaboration. Data extracted included left ventricular end systolic volume, LVEF, New York Heart Association classification, mitral regurgitation grade, and mortality rates.

**Results:**

Of the 37 articles included, 29 reported a mean volume reduction of >30%, while 8 reported a reduction <30%. A total of 4975 participants were included across all studies. Mean volume reduction was 43% in group 1 (>30%) and 28% in group 2 (<30%). The mean relative increase in LVEF for group 1 was 38%, which was larger compared to group 2 with 30%. Additionally, mean reduction in New York Heart Association class was 1.5 in group 1 and 1.2 in group 2. There was no difference in mitral regurgitation grade reduction between the 2 groups. Postoperative mortality within 30 days was 5.8% in group 1 vs. 5.2% in group 2.

**Conclusions:**

Both volume reduction groups showed improved LVEF and outcomes post-SVR, indicating the efficacy of SVR. The majority of studies achieved a volume reduction exceeding 30%, surpassing the 19% reported in the Surgical Treatment for Ischemic Heart Failure trial. Further meta-analysis can determine optimal volume reduction for enhanced cardiac function and outcomes.

## Highlights

• Thirty-seven studies: <30% vs. >30% volume reductions in surgical ventricular reconstruction.
• Compared cardiac function and mortality in <30% vs. >30% volume groups.
• Improved left ventricular ejection fraction in both volume groups.
• Surgical ventricular reconstruction improves cardiac function and patient outcomes.

## Introduction

Approximately 64 million people are diagnosed with heart failure (HF) worldwide.^1^ As life expectancy rises, the prevalence of HF is expected to increase.^2^ Ischemic cardiomyopathy (ICM), accounting for roughly 60% of HF cases, often involves anterior myocardial infarctions, which represent about 33% of all myocardial infarctions.^3^^,^^4^ Negative left ventricular (LV) remodeling occurs in around 30% of patients after acute myocardial infarction despite timely percutaneous coronary intervention and optimal guideline-directed medical therapy and occurs as a response to increased wall stress. Failure to normalize increased wall stress finally results in progressive LV dilation and contractile function deterioration.

In patients with severe LV systolic dysfunction and coronary artery disease suitable for intervention, coronary artery bypass grafting (CABG) is recommended as the primary revascularization strategy in patients with multivessel disease and acceptable surgical risk.^5^ Current guidelines of the European Society of Cardiology furthermore state that in experienced centers, surgical ventricular reconstruction (SVR) of the LV may be performed concomitant to CABG if HF symptoms are more predominant than angina and if myocardial scar and LV remodeling are present.^5^ By exclusion of akinetic and/or dyskinetic LV scar, SVR aims to restore the physiological volume and normal elliptical shape of the LV.^6^ If indicated, CABG and SVR can be performed in combination with mitral valve (MV) repair to address secondary mitral regurgitation (MR).

Although the Surgical Treatment for Ischemic Heart Failure (STICH) trial was designed and powered to evaluate the added value of SVR in ischemic HF patients, it showed unfavorable outcomes for SVR. First, the STICH trial investigators demonstrated that the addition of SVR to CABG did not result in a benefit in overall survival or survival free from cardiac hospitalization compared with CABG alone in patients with ICM.^7^ However, the results caused widespread discussion and were finally re-examined as the volume reduction achieved with SVR within the STICH trial was insufficient.^8^, ^9^, ^10^ Importantly, the probability of all-cause death was found to be significantly higher in patients that had a post-SVR LV end systolic volume index (LVESVI) of 60 mL/m^2^ or more. A similar trend in survival was correlated to the magnitude of decrease in LV volumes. Although not significant in the STICH population, a survival benefit was observed for patients in whom at least 30% reduction of LVESVI was achieved.^7^^,^^11^ In line with this, Isomura et al.^12^ showed that SVR in patients with a >33% volume reduction resulted in a higher survival rate. Moreover, positive effects of a 30% volume reduction on ventricular function and New York Heart Association (NYHA) classification were observed by the RESTORE group.^13^ We ther2efore performed a systematic review to evaluate the effect of the level of volume reduction (<30% vs. >30%) on LV function after SVR in patients with ICM. Secondary outcomes were the effects of volume reduction on ventricular function, NYHA classification, MR, and mortality.

## Methods

### Search Strategy

A systematic literature search was performed in PubMed, Embase, Web of Science, and The Cochrane Collaboration according to the Guidelines of the Preferred Reporting Items for Systematic Reviews and Meta-Analysis on April 16, 2021.^14^ A clinical librarian was consulted to assist with the search process. The complete search strategy can be found in Appendix A.

### Study Selection

Studies were included when the following characteristics were reported: SVR procedure performed in patients with ICM, left ventricular end systolic volume (LVESV), LVESVI, left ventricular ejection fraction (LVEF), NYHA class, MR grade, and mortality rates.

Articles were screened by 2 independent reviewers (H.O. and P.C.H.) on title and abstract. Divergences were resolved by consensus. We assessed whether studies described SVR in patients with ICM. Articles describing patients in whom SVR was performed for reasons other than ICM were excluded. Only articles written in English were included. A full text review was done by 2 reviewers (H.O. and P.C.H.) on selected studies. For the qualitative synthesis, studies were included if they met the inclusion criteria.

### Data Analysis

Data were extracted using Microsoft Excel (version 2019).^15^ The following data were extracted: author, year, title, study design, number of participants, mean age, inclusion criteria for SVR, type of intervention, follow-up period, method for measuring ventricular function, LVESVI or LVESV, LVEF, MR grade, NYHA class, and mortality. Results were divided into 2 groups: mean <30% volume reduction and mean >30% volume reduction. The outcomes were reported in tables, with the difference in LVEF between the 2 groups as the primary outcome and the differences in NYHA class, mortality, and MR grade as secondary outcomes. For each outcome, a weighted average was calculated using the formula: ∑(*n* in each study/Total *n* × Outcome value), where *n* represents the number of participants in each study for whom the specific outcome was measured, and Total *n* represents the total number of participants across all studies for that particular outcome. This method was applied to all outcomes, including LVEF, NYHA class, mortality, and MR grade, ensuring that the results were adjusted for the sample sizes reported in each study. Sensitivity analyses were conducted per outcome, excluding studies with fewer than 30 participants for each specific outcome.

### Quality Assessment

In the quality assessment, 2 specific tools tailored to the study designs of our included studies were employed: the Newcastle-Ottawa Scale for observational studies and the Risk of Bias 2 (RoB 2) tool for randomized studies.^16^^,^^17^ For observational studies, there was a focus on 3 main domains: selection, comparability, and outcome. In this process, a numeric score was assigned to each item within the Selection and Outcome categories, with the Comparability category allowing for a maximum of 2 scores per item. We then aggregated these scores to compute a total score for each study.

## Results

### Search Results

**Figure 1:**
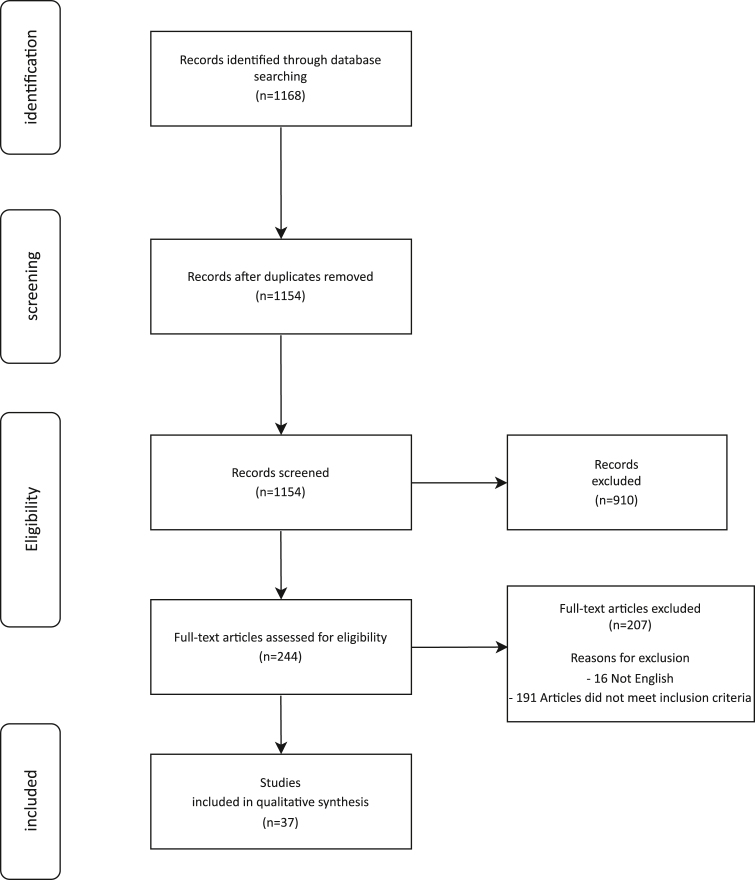
Flowchart.

The literature search yielded a total of 1168 articles. From these, *n* = 973 were retrieved from PubMed, *n* = 59 from Embase, *n* = 70 from Web of Science, and *n* = 66 from Cochrane. The search results are summarized in Figure 1. For the qualitative synthesis, 37 studies were included. Two randomized controlled trials and 35 observational studies were identified. The number of articles that reported the outcomes of interest were as follows: LVESVI and LVEF were reported in 37 articles, MR grade was reported in 8 articles, NYHA class was reported in 16 articles, and mortality was reported in 21 articles.

### Quality Assessment

Quality assessment was conducted for all studies in our systematic review. Both randomized controlled trials in our review, by Jones et al. and Aguiar Ribeiro et al., were evaluated using the RoB 2 tool. The study by Jones et al. was found to have a high risk of bias, whereas the study by Aguiar Ribeiro et al. was identified with some concerns regarding bias. The observational studies, assessed by the Newcastle-Ottawa Scale, displayed varying scores, reflecting diverse methodological quality. Detailed results of these assessments are presented in Tables B1 and B2 in Appendix B.

### Volume Reduction

The amount of volume reduction was calculated by using the mean preoperative and postoperative LVESVI data from 37 included articles. All articles reported mean LVESVI of the study participants. There was no data collection and analysis performed on patient-level related to the amount of volume reduction. In 2 publications, LVESV was reported and used to calculate the volume reduction.^18^^,^^19^ The amount of volume reduction achieved in the different studies are presented in Tables 1 and 2. The weighted average volume reduction was 43% in group 1 (*n* = 1562) and 28% in group 2 (*n* = 1488) (Tables 1 and 2). The lowest mean volume reduction was performed by Nomura et al.^47^ with a mean volume reduction of 16.7%. Di Donato et al. reported the highest mean volume reduction of 58.9%.^21^ The range of mean volume reduction in articles reporting >30% volume reduction (group 1) ranged between 34.2% and 58.9% (Table 1). Studies with a mean <30% volume reduction (group 2) ranged between 16.7% and 29.6% (Table 2). The sensitivity analyses in both groups demonstrated no notable changes in the weighted average volume reduction (Tables 1 and 2).

**Table 1:** Volume reduction >30% (group 1)

Author and year	N	*n*	Mean LVESVI (mL/m^2^)		Reduction in %	*p*-value
			Preoperative	Postoperative		
Athanasuleas, 1998^20^	8	8	132 ± 41.6	80.5 ± 30.3	39.0	<0.0002
Di Donato, 2001^21^	207	207	112 ± 64	46 ± 26	58.9	<0.0001
Suma, 2001^22^	50	50	113 ± 45.7	66.4 ± 28.4	41.5	<0.05
Athanasuleas, 2001^23^	439	185	109 ± 71	69 ± 42	36.7	<0.0001
Di Donato, 2001^24^	44	44	137 ± 70	77 ± 33	43.8	<0.0001
Menicanti, 2002^25^	46	46	98 ± 36	63 ± 22	35.7	0.001
Fuji, 2004^26^	14	14	78 ± 37	51 ± 34	34.6	<0.05
Kokaji, 2004^27^	16	15	115 ± 34	54 ± 14	53.0	<0.05
Di Donato, 2004^28^	30	30	144 ± 69	69 ± 40	52.1	0.001
Cirillo, 2004^29^	69	65	65.8 ± 29.8	37.7 ± 12	42.7	<0.0001
Menicanti, 2004^18^	108	108	170 ± 63^a^	107 ± 41^a^	37.1	0.000
Yamaguchi, 2005^30^	20	20	137 ± 24	65 ± 19	52.6	<0.05
Tulner, 2006^31^	21	21	186 ± 77	101 ± 50	45.7	<0.001
Ferrazzi, 2006^32^	85	65	89.6 ± 27.6	56.6 ± 34.5	36.9	<0.0001
Cirillo, 2006^33^	12	12	70.8 ± 12.6	42.4 ± 8.6	40.1	<0.05
Aguiar Ribeiro, 2006^34^	39	38	107 ± 19	65 ± 14	39.3	<0.001
Takeda, 2008^35^	72	72	111 ± 40	59 ± 25	46.8	<0.0001
Suma, 2009^36^	76	70	123.3 ± 38.9	74.0 ± 27.5	40.0	<0.001
Bové, 2009^37^	23	21	77 ± 17	40 ± 4	48.1	<0.05
Di Donato, 2009^38^	67	67	96 ± 36	57 ± 25	40.6	0.0001
Baravelli, 2010^39^	65	65	88 ± 33	57 ± 28	35.2	<0.0001
ten Brinke, 2010^19^	9	9	173 ± 77^a^	103 ± 40^a^	40.5	<0.001
Zhong, 2011^40^	40	40	117 ± 39	77 ± 31	34.2	<0.001
Dor, 2011^41^	117	101	96 ± 45	50 ± 21	47.9	0.01
Shudo, 2011^42^	21	21	123 ± 43	71 ± 31	42.3	<0.05
Skelley, 2011^43^	87	9	100.1 ± 50.0	57.7 ± 31.2	42.4	<0.05
Cho, 2012^44^	40	40	110.3 ± 35.9	57.8 ± 20.7	47.6	<0.0001
Cho, 2014^45^	102	99	104.1 ± 37.4	61.4 ± 21.9	41.0	<0.0001
Castelvecchio, 2021^46^	20	20	90.8	51.6	43.2	0.0002
		Total *n* = 1562			Weighted average = 43.0%	
Sensitivity analysis		Total *n* = 1392			Weighted average = 40.0%	

Abbreviations: LV, left ventricular; LVESV, left ventricular end systolic volume; LVESVI, left ventricular end systolic volume index.

a LVESV used for measuring LV volumes.

**Table 2:** Volume reduction <30% (group 2)

Author and year	N	*n*	Mean LVESVI (mL/m^2^)		Reduction in %	*p*-value
			Preoperative	Postoperative		
Athanasuleas, 2004^13^	1198	671	80.4 ± 51.4	56.6 ± 34	29.6	<0.001
Nomura, 2006^47^	26	26	112 ± 43	94 ± 47	16.7	0.0955
Menicanti, 2007^48^	1161	300	145 ± 64^a^	104 ± 50^a^	28.3	0.001
Ogawa, 2007^49^	4	4	135 ± 36	98 ± 28	27.4	NS
Jones, 2009^7^	501	161	83	67	19.3	NS
Calafiore, 2010^50^	44	44	67 ± 28	52 ± 19	22.4	NS
Isomura, 2011^12^	90	90	87.2 ± 31.8	63.1 ± 25.6	27.6	<0.0001
Zhong, 2011^51^	4	4	96 ± 22	63.1 ± 25.6	28.1	<0.05
		Total *n* = 1488			Weighted average = 28.0%	
Sensitivity analysis		Total *n* = 1266			Weighted average = 28.0%	

Abbreviations: NS, not significant; LV, left ventricular; LVESV, left ventricular end systolic volume; LVESVI, left ventricular end systolic volume index.

a LVESV used for measuring LV volumes.

### LVEF and NYHA Class

Mean relative LVEF increase between preoperative and postoperative LVEF ranged from 10% to 72% in group 1 and ranged between 5.1% and 33.4% in group 2 (Tables 3 and 4). In both groups, volume reduction was effective in improving NYHA class reduction. In group 1, the mean NYHA reduction ranged from 0.9 to 2.1 (Table 3). Mean NYHA reduction in group 2 ranged between 1.1 and 1.7 (Table 4). The weighted average LVEF increase was 38% in group 1 (*n* = 1741) and 30.0% in group 2 (*n* = 1586) (Tables 3 and 4). The weighted average NYHA class reduction was 1.5 in group 1 and 1.2 in group 2 (*n* = 1691) (Tables 3 and 4). The weighted averages for LVEF increase and NYHA class reduction remained effectively unchanged in the sensitivity analysis for both groups (Tables 3 and 4).

**Table 3:** Group 1: mean % LVEF increase and NYHA class decrease

Author and year	Volume reduction in %	Assessed for LVEF (*n*)	% of LVEF increase	*p*-value	Assessed for NYHA class (*n*)	NYHA class reduction	*p*-value
Athanasuleas, 1998^20^	39.0	8	72.0	<0.008			
Di Donato, 2001^21^	58.9	207	37.1	<0.0001			
Suma, 2001^22^	41.5	50	58.7	<0.05			
Athanasuleas, 2001^23^	36.7	354	34.5	<0.0001			
Di Donato, 2001^24^	43.8	44	35.3	<0.0001			
Menicanti, 2002^25^	35.7	46	10.0	0.11	38	1.6	<0.01
Fuji, 2004^26^	34.6	14	16.7	<0.05			
Kokaji, 2004^27^	53.0	15	62.1	<0.05			
Di Donato, 2004^28^	52.1	30	50.0	0.001			
Menicanti, 2004^18^	37.1	108	17.2	0.001	108	1.5	0.0001
Cirillo, 2004^29^	42.7	65	39.1	<0.0001	61	1.1	NM
Yamaguchi, 2005^30^	52.6	20	75.0	<0.0001			
Tulner, 2006^31^	45.7	21	33.3	0.007	21	2.0	<0.05
Ferrazzi, 2006^32^	36.9	65	55.8	<0.0001	65	1.6	<0.0001
Cirillo, 2006^33^	40.1	12	53.7	<0.05			
Aguiar Ribeiro, 2006^34^	39.3	38	52.5	<0.001	38	1.7	NS
Takeda 2008^35^	46.8	72	56.0	<0.0001	49	1.3	<0.0001
Suma,2009^36^	40.0	70	33.7	<0.001	42	2.1	NM
Bové, 2009^37^	48.7	21	44.4	<0.05	16	1.8	<0.001
Di Donato, 2009^38^	40.6	67	25.9	0.0001	67	1.2	0.0001
Baravelli, 2010^39^	35.2	65	31.0	<0.0001	65	0.9	<0.0001
ten Brinke, 2010^19^	40.5	9	27.8	<0.001	9	1.9	<0.01
Zhong, 2011^40^	34.2	40	19.2	<0.001			
Dor, 2011^41^	51.0	101	69.2	<0.001			
Shudo, 2011^42^	42.3	21	40.0	<0.05			
Skelley, 2011^43^	42.4	19	41.4	<0.05			
Cho, 2012^44^	47.6	40	20.7	<0.0001			
Cho, 2014^45^	41.0	99	25.9	<0.0001	99	1.5	0.0001
Castelvecchio, 2021^46^	43.2	20	56.1	0.0009			
		Total *n* = 1741	Weighted average = 38.0%		Total *n* = 678	Weighted average = 1.5	
Sensitivity analysis		Total *n* = 1561	Weighted average = 37.0%		Total *n* = 632	Weighted average = 1.4	

Abbreviations: NM, not mentioned; NS, not significant; NYHA, New York Heart Association; LVEF, left ventricular ejection fraction.

**Table 4:** Group 2: mean LVEF increase and NYHA class decrease

Author and year	Volume reduction in %	Assessed for LVEF (*n*)	% of LVEF increase	*p*-value	Assessed for NYHA class (*n*)	NYHA-class reduction	*p* -value
Athanasuleas,2004^13^	29.6	1118	33.4	<0.001	1198	1.2	NM
Nomura, 2006^47^	16.7	26	0.0	0.95	26	1.7	<0.0001
Menicanti, 2007^48^	28.3	300	27.4	<0.001	463	1.1	<0.001
Ogawa, 2007^49^	27.4	4	29.4	NS	4	1.3	NM
Jones, 2009^7^	19.3		NM	NM		NM	NM
Calafiore, 2010^50^	22.4	44	5.1	NM			
Isomura, 2011^12^	27.6	90	15.5	0.0004			
Zhong, 2011^51^	28.1	4	20.8	NM			
		Total *n* = 1586	Weighted average = 30.0%		Total *n* = 1691	Weighted average = 1.2	
Sensitivity analysis		Total *n* = 1552	Weighted average = 30.0%		Total *n* = 1661	Weighted average = 1.2	

Abbreviations: NM, not mentioned; NS, not significant; NYHA, New York Heart Association; LVEF, left ventricular ejection fraction.

### MR Grade

MR grade was reported in 3 articles in group 1 and in 5 articles in group 2 (Tables 5 and 6). In group 1, mean MR grade reduction ranged between 0.4 and 2.2 (Table 5). The highest MR grade reduction reported in group 2 was 1.7 (Table 6). The weighted average MR grade decrease was 1.4 in group 1 (*n* = 255) and 1.4 in group 2 (*n* = 370) (Tables 5 and 6). No substantial alteration was observed in the weighted MR grade reduction in both groups in the sensitivity analyses (Tables 5 and 6).

**Table 5:** Group 1: mean MR decrease

Author and year	Volume reduction in %	Assessed for MR (*n*)	MR decrease	*p*-value
Menicanti, 2004^18^	37.1	108	2.2	0.0001
Tulner, 2006^31^	45.7	21	1.0	0.001
Bové, 2009^37^	48.7	21	1.8	<0.05
Baravelli, 2010^39^	35.2	65	0.5	<0.0001
Cho, 2012^44^	47.6	40	0.4	NM
		Total *n* = 255	Weighted average = 1.4	
Sensitivity analysis		Total *n* = 213	Weighted average = 1.3	

Abbreviations: MR, mitral regurgitation; NM, not mentioned;.

**Table 6:** Group 2: mean MR decrease

Author and year	Volume reduction in %	Assessed for MR (*n*)	MR decrease	*p*-value
Nomura, 2006^47^^,^^48^	16.7	26	1.7	<0.0001
Menicanti, 2007^48^	28.3	300	1.5	<0.001
Calafiore, 2010^50^	22.4	44	0.4	NM
		Total *n* = 370	Weighted average = 1.4	
Sensitivity analysis		Total *n* = 344	Weighted average = 1.4	

Abbreviations: MR, mitral regurgitation; NM, not mentioned;.

### Mortality

In group 1, the highest percentage of >30-day mortality was 35.6% and the lowest was 2.6%. In this group, <30-day mortality ranged from 4.3% to 16.7% (Table 7). The <30-day mortality in group 2 ranged between 3.6% and 11.2% (Table 8). The weighted average mortality <30 days postoperative in group 1 was 5.8% (*n* = 1212) and >30 days postoperative weighted average mortality was 5.3% (Table 7). The weighted average mortality <30 days postoperative for group 2 was 5.2% (*n* = 2930) (Table 8). The sensitivity analysis indicated that the weighted average mortality rates remained largely unchanged (Tables 7 and 8).

**Table 7:** Group 1: mortality

Author and year	Volume reduction (%)	N	Follow-up time	Mortality <30 d postoperative (%)	Mortality >30 d postoperative (%)
Di Donato, 2001^21^	58.9	207	39 ± 19 mo		13.0
Athanasuleas, 2001^23^	36.7	439	18 mo	6.6	
Kokaji, 2004^27^	53.0	16	43.7 ± 28.7 mo	4.4	
Menicanti, 2004^18^	37.1	108	6 y	16.7	
Cirillo, 2004^29^	42.7	69	1.9 ± 1.3 y	4.3	7.5
Aguiar Ribeiro, 2006^34^	39.3	39	2 y		2.6
Takeda, 2008^35^	46.8	72	3.3 ± 2.4 y	2.8	
Suma, 2009^36^	40.0	76	NM	7.9	
Bové, 2009^37^	48.1	23	20.9 ± 10.7 mo	13.0	
Di Donato, 2009^38^	40.6	67	30 ± 21 mo	5.9	
ten Brinke, 2010^19^	40.5	9	6 mo	10.3	
Skelley, 2011^43^	42.4	87	22.4 mo	4.6	35.6
		Total N = 1212		Weighted average = 5.8%	Weighted average = 5.3%
Sensitivity analysis		Total N = 1164		Weighted average = 5.7%	Weighted average = 5.5%

Abbreviation: NM, not mentioned.

**Table 8:** Group 2: mortality

Author and year	Volume reduction in %	N	Follow-up time	Mortality <30 d postoperative in %	Mortality >30 d postoperative in %
Athanasuleas, 2004^13^	29.6	1198	5 y	5.3	
Nomura, 2006^47^	16.7	26	5 y	3.6	
Menicanti, 2007^48^	28.3	1161	56 ± 48 mo	4.9	
Jones, 2009^7^	19.3	501	48 mo	5.2	
Calafiore, 2010^50^	22.4	44	77 ± 50 mo	11.2	
		Total N = 2930		Weighted average = 5.2%	
Sensitivity analysis		Total N = 2904		Weighted average = 5.2%	

## Discussion

A systematic review was performed to compare a less than 30% with a more than 30% LV volume reduction on ventricular function, NYHA, MR, and mortality in patients with ICM that underwent SVR with or without CABG. A total of 37 articles were identified; 29 studies reported a >30% volume reduction (group 1), and 8 studies reported a <30% reduction (group 2). Mean volume reduction and relative LVEF increase were calculated by taking the difference between the mean preoperative and postoperative values and dividing it by the preoperative values. Reduction in mean NYHA class and MR grade were calculated by computing the difference between preoperative and postoperative values. Additionally, weighted averages were calculated for each outcome based on the total number of patients across the studies.

The cut-off value of 30% was based on international data of more than 5000 patients showing favorable effects on patient outcomes with a 30% or more volume reduction.^52^, ^53^, ^54^, ^55^ In both groups, beneficial effects of volume reduction were identified on LVEF. However, a notable difference was observed between the groups in terms of LVEF improvement. In group 1, the weighted average LVEF increase was higher at 38%, with individual studies reporting increases ranging from 10% to 75%. All studies besides that of Menicanti et al. showed a significant difference between preoperative and postoperative LVEF (*p*-value <0.05) in group 1. In contrast, group 2 showed a lower weighted average LVEF increase of 30%, with individual studies reporting increases ranging from 0 to 33.4%. Notably, only 3 studies in this group showed significant improvements in LVEF, with volume reductions of 29.6, 28.3, and 27.6%. These reductions are close to the 30% cut-off. Among the other publications (n = 4, one paper did not mention LVEF difference), 2 studies reported no significant LVEF increase, and the other 2 studies did not mention significance. In group 2, 3 of the 8 articles had a volume reduction less than 25% (16.7, 19.3%, and 22.4%). One of these articles showed no significant difference in LVEF; one study did not mention LVEF, and the other study did not mention significance. The sensitivity analysis on the amount of volume reduction and LVEF showed no meaningful changes, supporting the robustness of the findings.

Secondary outcomes included NYHA classification, MR grade, and mortality. Both MR grade reduction and NYHA classification improvement were observed in both groups; however, a greater reduction in the weighted NYHA score was reported in group 1 compared to group 2. No notable differences in MR grade reduction were identified between the groups. Regarding mortality, a modest difference was observed in <30-day mortality, with higher rates in group 1 compared to group 2. Sensitivity analyses for the secondary outcomes revealed no substantial differences, reinforcing the stability of these findings. However, definitive conclusions regarding these secondary outcomes cannot be drawn, as no formal statistical analysis was conducted to evaluate these differences.

The importance of >30% volume reduction performed by SVR has been known before the start of the STICH trial. Based on the international data, the STICH trial investigators considered a volume reduction of at least 30% to be sufficient.^52^, ^53^, ^54^, ^55^, ^56^ However, the STICH trial did not meet the criteria of adequate volume reduction and achieved an average volume reduction of only 19%.^7^ To the best of our knowledge, Jones et al. did not report why the >30% volume reduction criteria of the STICH trial were not met. There may be several explanations for not meeting the STICH criteria: only 33% (*n* = 161 of 490) of the patients underwent preoperative and postoperative LVESVI measurement, and 19% of the patients had an ultrasound measurement despite the requirement for a cardiovascular magnetic resonance measurement. The available literature and the results of this review may suggest that the STICH trial contradicts the evidence available on the benefits of SVR.^13^^,^^48^^,^^57^ In one of the included studies with 1198 participants, SVR increased LVEF significantly from 29.6% ± 11.0% preoperatively to 39.5% ± 12.3% (*p* < 0.001) postoperatively.^13^ Isomura et al.^12^ performed a retrospective analysis on 90 patients and reported potential benefit in cardiac function if volume reduction occurred by SVR. In this study, a volume reduction of >33% resulted in improved LVEF. The group with >33% volume reduction had a significantly higher postoperative LVEF compared to the group with 13% and 15% volume reduction. In our review, we also observed favorable effects of >30% volume reduction on LVEF, with a higher weighted average LVEF in group 1 compared to group 2. These findings reinforce the notion that SVR could be an effective treatment for improving LVEF. The results of this review presented that the cut-off value of 30% volume reduction is not a strict cut-off. Nevertheless, outcomes and literature reported that a volume reduction around and above 30% is beneficial for LVEF.

Ischemic dilated cardiomyopathy is associated with MR. In this review, 8 studies were identified that included the outcome on MR. SVR is often performed concomitant with MV repair.^52^, ^53^, ^54^, ^55^, ^56^^,^^58^ Despite the fact that there are studies showing the benefits of SVR on MR, we were unable to demonstrate a difference in MR grade reduction in the >30% volume reduction and <30% volume reduction group.^47^^,^^59^

In the present review, mortality rates were compared in patients with a <30% and >30% ventricular volume reduction. We observed a modest difference in weighted <30-day postoperative mortality, with group 1 (>30% volume reduction) showing slightly higher rates than group 2 (<30% volume reduction). The lack of significant mortality differences between these groups may be attributed to extensive baseline ventricular remodeling, which could have limited the impact of ventricular reconstruction.^60^^,^^61^ Furthermore, other studies have reported favorable outcomes in general regarding SVR and its impact on mortality. A study performed by Sartipy et al.^57^ reported a 5-year survival of 68% in patients undergoing SVR. A survival rate at 3 years of 83% and operative mortality of 5.4% in patients undergoing SVR (with mean volume reduction of 37.7%) concomitant with CABG was reported by Di Donato et al.^62^ Athanasuleas et al.^13^ reported a 30-day mortality of 5.3% when performing volume reduction (mean 29.6%) in 671 patients. In addition to the volume reduction itself, LV shape is likely to influence the mortality rate of patients with ICM. In a retrospective study of 401 patients, di Mauro et al. examined whether volume or shape improved patient outcomes when treating patients with ICM.^63^ The results of the study showed that a volume reduction with a more conical shape resulted in a decrease of 30-day mortality and better 10-year freedom of cardiac death and events than volume reduction alone.^63^

NYHA score improvement was highest in group 1 at a volume reduction of 40% with a mean NYHA class reduction of 2.1. In group 2, NYHA improvement was highest at a volume reduction of 16.7% with a significant NYHA class reduction of 1.7. A small difference in weighted NYHA class reduction was noted between the groups; however, this difference was minimal, clinically irrelevant, and therefore insufficient to draw definitive conclusions.

This study was subject to some limitations. A major limitation of our review is that most included studies are observational, with the exceptions of 2 randomized controlled trials: the STICH trial and the study by Aguiar Ribeiro’s team. Randomized controlled trials, if done correctly, provide unbiased effect estimates and enable causal inference. The predominance of observational studies, combined with the fact that most studies involved both SVR concomitant with mitral valve repair and/or CABG, complicates the ability to accurately determine which of the procedures contributed to the observed outcomes. Additionally, there was considerable heterogeneity among the included studies. Most studies differed in their inclusion criteria, follow-up time, and diagnostics for measuring cardiac function. Furthermore, the total number of patients assessed for each outcome varied between group 1 and group 2 across studies, limiting the validity of direct comparisons between the groups, particularly for the mortality outcome, where this discrepancy was most pronounced. Moreover, the studies by Menicanti et al. and Athanasalues et al. are included in both volume reduction groups, potentially leading to biased results. Since the mean LVESVI was reported in all included articles and there was no review of the amount of volume reduction on patient level, it could be possible that a part of the LVESV reduction from the included population per study was above or below the cutoff value of 30%. In addition, the validity of MR decrease is also a limitation in our study. In the different studies, it was not always evident whether MV surgery was performed, nor do we know in which patients MV surgery was performed because we did not have individual patient data from the studies. A meta-analysis could be supportive to provide a cut-off value of a volume reduction on cardiac function and patient outcomes.

## Conclusion

This systematic review, performed according to the Preferred Reporting Items for Systematic Reviews and Meta-Analysis guidelines, provided an extensive overview of the existing literature on the extent of volume reduction after SVR and its effect on cardiac function and patient outcomes. This review demonstrates that a larger LV volume reduction is associated with a greater improvement in LVEF, greater reduction in heart failure symptoms, and higher <30-day mortality. However, whether a cut-off of >30% volume reduction is appropriate remains unsettled, because a substantial number of patients in the <30% group had reductions close to 30%. A meta-analysis with individual patient data would provide a more accurate analysis.

## CRediT Authorship Contributions

All listed authors qualify for authorship based on their substantial contributions: study concept and design (Reinier Zandbergen, Harun Osmani, and Paulien Christine Hoefsmit), acquisition of data (Harun Osmani, Paulien Christine Hoefsmit, and George Louis Burchell), analysis and interpretation of data (Reinier Zandbergen, Jolanda Kluin, Patrick Klein, Harun Osmani, Paulien Christine Hoefsmit, and Romy R.M.J.J. Hegeman), drafting of the manuscript (Reinier Zandbergen, Jolanda Kluin, Harun Osmani, George Louis Burchell, and Paulien Christine Hoefsmit), and critical revision (Reinier Zandbergen, Jolanda Kluin, Patrick Klein, Harun Osmani, Paulien Christine Hoefsmit, and Romy R.M.J.J. Hegeman). All authors have read and approved the final manuscript.

## Ethics Statement

Not applicable.

## Funding

The authors have no funding to report.

## Disclosure Statement

The authors report no conflict of interest.

## Availability of Data and Materials

The full search strategy is included as supplementary file.
